# Renal angina: concept and development of pretest probability assessment in acute kidney injury

**DOI:** 10.1186/s13054-015-0779-y

**Published:** 2015-02-27

**Authors:** Lakhmir S Chawla, Stuart L Goldstein, John A Kellum, Claudio Ronco

**Affiliations:** Department of Medicine, Division of Intensive Care Medicine and Division of Nephrology, Veterans Affairs Medical Center, 50 Irving Street, Washington, DC 20422 USA; Department of Anesthesiology and Critical Care Medicine, George Washington University Medical Center, 900 23rd Street, Washington, DC 20037 USA; Center for Acute Care Nephrology, Cincinnati Children’s Hospital Medical Center, 3333 Burnet Avenue, MLC 7022, RILF2, Cininnati, OH USA; Department of Critical Care Medicine, Center for Critical Care Nephrology, The CRISMA (Clinical Research, Investigation, and Systems Modeling of Acute Illness) Center, University of Pittsburgh School of Medicine, 3550 Terrace Street, Pittsburgh, PA 15261 USA; Department of Nephrology, Dialysis & Transplantation, International Renal Research Institute, San Bortolo Hospital, Via Bertesina, Vicenza, 36100 Italy

## Abstract

The context of a diagnostic test is a critical component for the interpretation of its result. This context defines the pretest probability of the diagnosis and forms the basis for the interpretation and value of adding the diagnostic test. In the field of acute kidney injury, a multitude of early diagnostic biomarkers have been developed, but utilization in the appropriate context is less well understood and has not been codified until recently. In order to better operationalize the context and pretest probability assessment for acute kidney injury diagnosis, the renal angina concept was proposed in 2010 for use in both children and adults. Renal angina has been assessed in approximately 1,000 subjects. However, renal angina as a concept is still unfamiliar to most clinicians and the rationale for introducing the term is not obvious. We therefore review the concept and development of renal angina, and the currently available data validating it. We discuss the various arguments for and against this construct. Future research testing the performance of renal angina with acute kidney injury biomarkers is warranted.

## Introduction

The use of advanced diagnostics (that is, beyond history and physical examination) forms a cornerstone of modern medicine. For any given diagnostic test, the context of that test is a critical component for the interpretation of the results. This context is referred to as pretest probability and forms the basis for the interpretation of any diagnostic test. For instance, if a young healthy pregnant woman presented for a medical examination and had a very elevated prostate specific antigen value, the patient’s physician can safely dismiss this as a specious result. However, if the patient was a 70-year-old man with nocturia with the same elevated prostate specific antigen value, the clinician would arrive at a different conclusion. While this is an extreme example, it demonstrates the simple rule that all clinicians follow: the value of any diagnostic study without clinical context is minimized. The assessment of pretest probability for any diagnosis is informed by all of the core aspects of a medical examination (that is, history, physical examination, and all available medical information). The integration of these data then informs a differential diagnosis that guides the selection and interpretation of diagnostic studies.

In the past 30 years, protein biomarkers have become important sources for development of diagnostic tests. The biomarker that many hold up as a gold standard for comparison is troponin for the diagnosis of acute coronary syndrome (ACS) and myocardial infarction. The use, accuracy, and utilization of troponin are well appreciated and have been reviewed extensively [1]. The typical utilization for troponin and other ACS biomarkers is in the setting of patients who present with angina pectoris and angina pectoris-type symptoms (for example, chest pain, dyspnea, referred visceral pain). When used in this venue, troponin serves as an outstanding biomarker, which helps to either rule in or rule out significant ACS. This performance is due to two facts: troponin is a good biomarker with a high sensitivity and specificity; and the pretest probability of a patient with angina to have ACS is high. However, when troponin is utilized in a less selective fashion, in patients who have a lower pretest probability of ACS, the performance of this much heralded biomarker deteriorates [2,3]. For example, the sensitivity and specificity of fourth-generation troponin in patients with angina pectoris are 88% and 88%, respectively [4]. When this same assay was used in critically ill patients without typical anginal symptoms, only one-half of the patients with an elevated troponin were found to have any type of ACS [3]. In this study, the false positive elevations were attributed to other causes (that is, sepsis, renal failure). Studies such as these demonstrate that for any biomarker, even one as good as troponin, clinical context and pretest probability assessment remain paramount.

## Angina

### Deconstructing angina pectoris

The differential diagnosis for chest pain is taught to all medical students early on in their medical education. The concept of chest pain and its character (sharp versus squeezing), onset (sudden versus gradual), and location (substernal) are imparted to trainees. These signs and symptoms are then placed into context with the well-known risk factors of ACS (family history, diabetes, hypertension, elevated cholesterol, and so forth). With these data in hand, a clinician is able to develop a level of suspicion for the presence or absence of ACS. This clinical approach has been instrumental in the evaluation of patients with ACS, and has been further improved with the appropriate use of sensitive and specific biomarkers (for example, troponins and before that creatinine kinase).

As detailed above, the approach to a patient with suspicion for ACS has been well constructed and validated, but the same is not true for patients with acute kidney injury (AKI). Only recently has AKI developed a consensus definition and there remains a tendency to diagnose the disease late in its course [5,6]. To improve the time to diagnosis, multiple AKI biomarkers have been discovered, assessed, and validated. These biomarkers have been tested in scores of studies and found to be good diagnostic markers [7-10]. However, many of these biomarkers failed to achieve troponin-like performance. For purposes of comparison, most diagnostic tests can be assessed using a receiver operating characteristic (ROC) assessment. The range for this test is an area under the curve (AUC) of 0.50 to 1.00. At a ROC AUC value of 0.50, the test has maximum uncertainty and is equivalent of flipping a coin. At a ROC AUC value of 1.00, the test performs with perfect diagnostic capacity. The fourth-generation troponin assays have a ROC AUC performance of 0.89 to 0.91 in patients with angina pectoris, which is excellent [4]. In most cases, many of the current AKI biomarkers typically have a performance in the range of 0.65 to 0.84 when tested using current study designs [8-11].

However, ROC curves are influenced by the severity of illness found in the population under study. We considered that some of the underperformance was in part not due to poor biomarkers, but rather due to inadequate assessment of context and pretest probability. In fact, most of these studies wherein the AKI biomarkers performed poorly were in patient cohorts with heterogeneous disease processes, while the performance of AKI biomarkers was much better in more homogeneous populations (for example, postoperative cardiac surgery in children) [8-10]. As shown earlier, the performance of troponin drops substantially when used in heterogeneous populations. We thus developed and proposed the concept of renal angina (RA) in order to better assess pretest probability of AKI to inform the use and interpretation of AKI biomarkers [12].

### Constructing renal angina

With this goal in mind, we analyzed the two core components of angina pectoris – risk factors plus symptomology – to develop the parallel of RA. The risk factors for AKI are well documented in various patient populations (Table 1) [5,12], but AKI does not have any visceral symptoms. Put another way, AKI does not hurt. We therefore could not use flank pain or dysuria to develop the symptomology of AKI. Instead, we utilized the clinical signs that are used to assess AKI: urine output, changes in serum creatinine, and percent fluid overload. We hypothesized that very small changes in serum creatinine, short periods of oliguria, and fluid overload could be used in place of visceral anginal symptoms. We then used combinations of risk factors and modest changes in serum creatinine, urine output, and fluid overload to develop the definition of RA.

**Table 1:** **Acute kidney injury risk factors**

	**Adults**	**Children**
Susceptibilities	Advanced age	Very premature neonates
	Congestive heart failure	Heart failure
	Hypertension	Stem cell transplant
	Diabetes mellitus	
	Chronic kidney disease	
Exposures	Volume depletion	Volume depletion
	Cardiopulmonary bypass	Cardiopulmonary bypass
	Nephrotoxin exposure	Nephrotoxin exposure
	Mechanical ventilation	Mechanical ventilation
	Sepsis	Sepsis
	Vasopressors	Vasopressors

**Figure 1:**
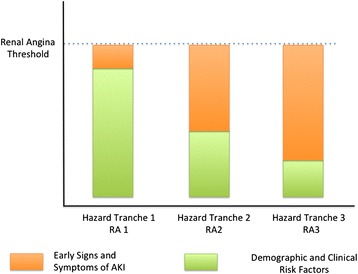
**Hazard tranches to incorporate an operating concept into reginal angina.** Each hazard tranche (HT) has equivalent net risk for the development of acute kidney injury (AKI). RA, reginal angina.

The premise of this approach is as follows. Patients who have many risk factors for ACS do not need to show much chest pain in order for a clinician to become suspicious that ACS is present. An example would be an obese diabetic male with mild heartburn symptoms. The same symptomology in an Olympic athlete in her prime would engender less suspicion of ACS with the same level of syptomology. Inherent in the angina pectoris approach is the notion that the presence of multiple ACS risk factors lowers the threshold for generating suspicion of ACS. We thus developed three hazard tranches (HT) to incorporate this operating concept into RA (Figure 1). In our proposal, each HT has equivalent net risk for the development of AKI. HT1 has multiple AKI risk factors and requires few signs to achieve the RA threshold. HT2 is comprised of patients with some AKI risk factors and thus requires slightly more signs to achieve the RA threshold. HT3 includes patients with few risk factors for AKI and thus requires more definitive signs to achieve the RA threshold.

The inherent hypothesis in this approach is that HT1, HT2, and HT3 would have similar risk profiles for the development of severe AKI, defined by Kidney Disease: Improving Global Outcomes stages II to III. We proposed criteria for both children and adults [12]. The second hypothesis of RA is that, like angina pectoris, it should be very sensitive and have a high negative predictive value (NPV). The third hypothesis of RA is that when AKI biomarkers are used in patients with RA, the diagnostic performance of those biomarkers will improve, similar to troponin use in angina pectoris.

## Renal angina risk prediction

**Figure 2:**
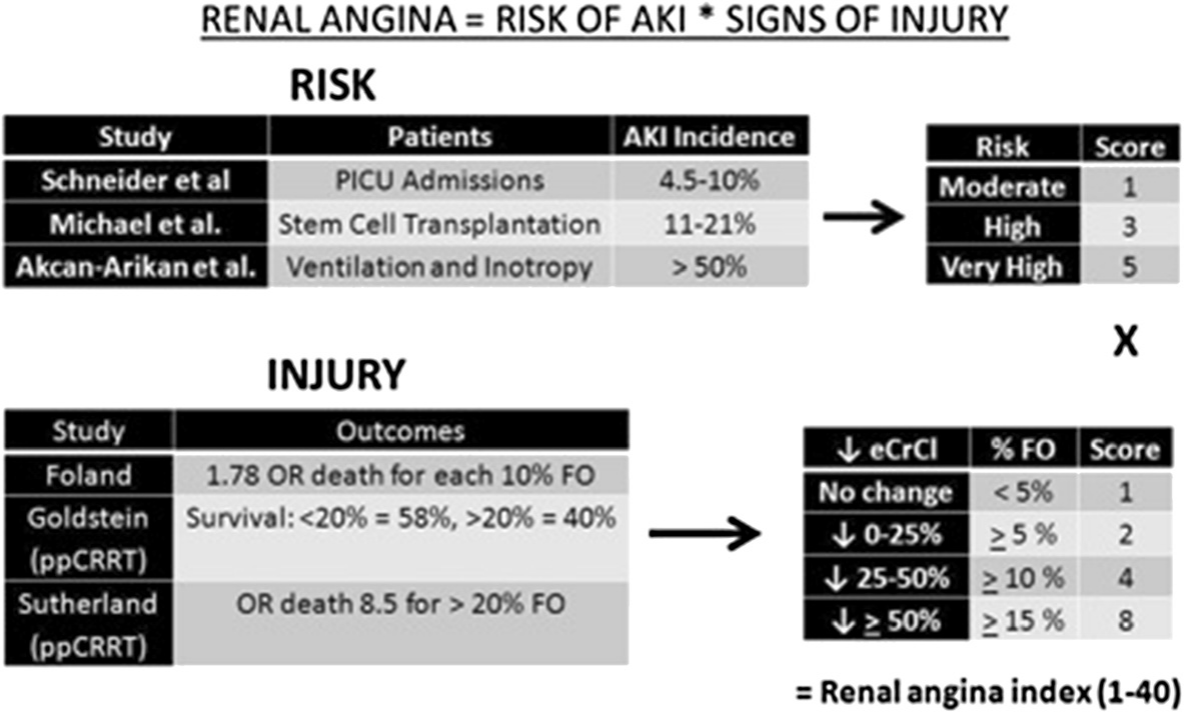
**Renal angina index for children.** The renal angina index establishes point values for both risk tranche and injury threshold to use in computing a composite score. The integer values assigned for each risk tranche (that is, 1, 3, 5) are derived directly by comparing cohorts extracted from established pediatric acute kidney injury (AKI) data in an epidemiologic study. The integer values given for injury are denoted as two-exponent to signify doubling of injury for incremental increases in creatinine or fluid overload on admission. The computed index ranges from 1 to 40. Through sensitivity analyses, cutoff value ≥8 has been established as fulfillment of renal angina. eCrCl, estimated creatinine clearance; FO, fluid output; OR, odds ratio; PICU, pediatric ICU; ppCRRT, positive-pressure continuous renal replacement therapy. Adapted with permission from [13].

Since the initial RA proposal in 2010, RA has been assessed in four pediatric cohorts and one adult cohort [13]. In the pediatric cohorts, a renal angina index (RAI) was developed and validated to operationalize the bedside use of RA (Figure 2). In a recent study, Basu and colleagues assessed four cohorts of critically ill pediatric patients and assessed the performance of RA using the RAI [13]. They found that RA could be operationalized for use in the pediatric ICU population to predict severe AKI 3 days after ICU admission. The performance of the RAI in each of these pediatric cohorts was remarkably consistent with a risk prediction performance of ROC AUC of 0.74 to 0.81 (Table 2) and a NPV of 92 to 99%.

**Table 2:** **Diagnostic performance of renal angina**

	**Peds1**	**Peds2**	**Peds3**	**Peds4**	**Adults [** 14 **]**
*n*	144	118	108	214	506
Sensitivity (%)	75	58	91	93	92
Specificity (%)	73	90	71	36	62
Positive predictive value (%)	40	39	26	18	16
Negative predictive value (%)	92	95	99	97	99
ROC AUC^a^	0.77	0.74	0.81	0.80	n/a

Pediatric cohorts as reported by Basu and colleagues [13]. n/a, not available. ^a^Receiver operator characteristics area under the curve (ROC AUC) for the renal angina index.

RA has been assessed in one large cohort of critically ill adult patients. In a study of over 500 adult ICU patients in Italy, Cruz and colleagues assessed the capacity of RA to predict severe AKI [14]. Their results are consistent with the performance of RA in children. The authors found that RA was strongly associated with the development of AKI, and that the sensitivity was high (92%) with a high NPV (99%) (Table 3). In both the adult and pediatric cohorts, the NPV was high (92 to 99%) (Table 3). In addition, Cruz and colleagues assessed the performance of each of the HTs [14]. They found the performance to indicate that the HTs perform similarly, and the sensitivity is high with an excellent NPV.

**Table 3:** **Performance of renal angina within hazard tranches**

	**HT1**	**HT2**	**HT3**	**Entire cohort**
Sensitivity (%)	100	92	100	92
Specificity (%)	11	66	76	62
Positive predictive value (%)	17	14	19	16
Negative predictive value (%)	83	86	100	99

Data from Cruz and colleagues [14]. Hazard tranches: HT1, patients at very high risk; HT2, patients at high risk; HT3, patients at moderate risk.

Overall, the preliminary studies of RA endorse the notion that this approach can be operationalized at the bedside. Moreover, the intertranche consistency and the consistent finding of a high NPV suggest that absence of RA can be used to rule out AKI. In addition, the fact that the pediatric and adult cohorts perform similarly offers further evidence of the pediatric Risk, Injury, Failure, Loss, End-staging system being well calibrated to the adult Risk, Injury, Failure, Loss, End-staging AKI criteria, and that this pretest probability approach is relevant in AKI to both adults and children. Since the disease epidemiology leading to AKI is similar in children and adults (sepsis, post-bypass, nephrotoxic medication injury) and AKI is independently associated with increased mortality in children and adults, it is in fact not surprising that the RA construct and AKI definitions yield similar results. Most novel AKI biomarkers have been tested initially in children, since they provide a clinical cohort less confounded by chronic disease. We support the recent recommendation from the American Society of Nephrology Acute Kidney Injury Advisory Committee to include adolescents in adult AKI [15].

## The case against renal angina

Of course, like any new paradigm, there are sensible arguments against introducing the term and the concept of RA. First and foremost, the term angina is most commonly associated with angina pectoris and many clinicians will associate it with ischemia. However, the term is not specific to the heart or to ischemia. Various forms of angina have been described, notably Ludwig’s angina and Vincent’s angina. Wilhelm Friedrich von Ludwig first described Ludwig’s angina in 1836, which is an infection in the floor of the mouth. According to Merriam-Webster, the term Vincent’s angina was first used in 1903 and was often used interchangeably with the term trench mouth. Neither Ludwig’s angina nor Vincent’s angina is associated with chest pain, arterial stenosis, or ischemia *per se* [16-19]. Both of these forms of angina are still currently used in the medical literature [18-20]. The notion that angina is only associated with the current cardiac vernacular is thus not supported by its historical and current use.

Second, even if the term angina can be placed into the above context it may be unfamiliar to many clinicians. However, this is already changing – the term RA is now well established in the literature. The original paper published in 2010 [12] has been cited over 50 times [7,13,21-37] (edited bibliography). In addition, validation of the pediatric RAI has been published in *Kidney International* [13] and validation of the adult RA proposal was published recently in *Clinical Journal of the American Society of Nephrology* [14].

Third, it is well known that AKI occurs most commonly in the critically ill, and often in this population as part of multiple organ failure. Such patients already have the AKI equivalent of chest pain. However, AKI is still common in patients without such severity of illness, and in fact these low-risk patients exhibit a larger relative hazard when they develop AKI [38]. Patients outside the ICU are similarly low risk and yet the importance of identifying AKI in this population is not in question. Indeed, the purpose of RA is to provide a framework for risk stratification that incorporates static and dynamic factors so the clinician can make sensible decisions regarding risk stratification.

## Conclusions

The concept of RA to operationalize pretest probability assessment in AKI appears to have good performance metrics. Importantly, in children the combination of RA and AKI biomarkers has excellent diagnostic performance and this combination strategy should be the primary focus of RA utilization. Future research will need to assess the performance of RA with AKI biomarkers in adults. In addition, it is important to recognize that RA will need to be adjusted and recalibrated as new information becomes available. New diseases and nephrotoxins will evolve over the years and other important risk factors for AKI will be discovered. These new facts will need to be appropriately incorporated into the RA schema.

## Abbreviations

ACS: Acute coronary syndrome
AKI: Acute kidney injury
AUC: Area under the curve
HT: Hazard tranche
NPV: Negative predictive value
RA: Renal angina
RAI: Renal angina index
ROC: Receiver operating characteristic
